# The Impact of Personal Protective Equipment on Healthcare Workers on COVID-19 Duty in a Tertiary Care Hospital in South India

**DOI:** 10.7759/cureus.41910

**Published:** 2023-07-14

**Authors:** Raymond Haward, Ridhima G, Meenakshi Kalyan

**Affiliations:** 1 Medical School, Vydehi Institute of Medical Science and Research Centre, Bangalore, IND; 2 Internal Medicine, Vydehi Institute of Medical Science and Research Centre, Bangalore, IND

**Keywords:** healthcare workers, ppe shortage, sars-cov-2 (severe acute respiratory syndrome coronavirus -2), covid-19, health care workers, personal protective equipment

## Abstract

Context

The proper usage of personal protective equipment (PPE) must be prioritised for health care workers (HCWs), where shortages and prolonged use of personal protective equipment can threaten safety in essential health services.

Aims

To evaluate the effect of personal protective equipment on the health and well-being of HCWs, physicians, nurses, and technicians on duty for COVID-19 rotational postings.

Settings and design

This cross-sectional study was done by simple random sampling.

Methods and materials

This study was conducted at a tertiary care centre in South India to assess the utilisation of personal protective equipment (PPE) during the second wave of COVID-19. A physical questionnaire was distributed to a total of 266 healthcare workers, aged 20 to 50, who had worked for a minimum of three consecutive days between May and August 2021. The objective of the study was to evaluate the effectiveness of PPE use among healthcare workers during the second wave of the COVID-19 pandemic.

Statistical analysis

The data analysis in this study was conducted using IBM Statistical Package for Social Sciences (SPSS) version 19 (IBM Corp., Armonk, New York). The mean and standard deviation, or median, were used to present continuous variables, while frequency and percentage were used to present categorical variables. Furthermore, the minimum sample size required for this study was calculated to be 246 participants.

Results

The survey included 266 healthcare workers. The mean+/-SD of age was 28.18+/-5.64 and consisted of females (54.51%) and males (45.48%). The postings were in emergency (13.15%), intensive care unit (30.82%), and ward (56.01%), respectively. The HCWs who used PPE for four to seven days reported more symptoms than those who used it for one to three days. Discomforts experienced while wearing PPE were chest suffocation (49.62%), difficulty in performing intubation (36.09%), difficulty in seeing clearly (68.79%), dizziness (49.62%), excessive sweating (75.56%), micturition desire (52.63%), nausea (42.48%), retro-auricular pain (56.76%), stomach burns (27.44%), and thirst or dry throat (78.57%). The symptoms suffered after doffing were tiredness (69.17%), dry mouth (67.29%), dizziness (43.60%), headache (55.63%), chest suffocation (36.46%), dry skin (57.14%), reduced ability to concentrate (48.12%), dark-coloured urine (55.63%), reduced alertness (42.48%), and stomach burns (28.94%). The first thoughts after doffing were to drink water (68.42%), eat something (36.09%), clean yourself (61.27%), urinate (33.08%), and have some rest (29.32%), respectively. 81 (30.45%). The HCWs suffered skin injuries while wearing gloves. The time for restoring after a shift was 12 hours (37.59%), 24 hours (34.21%), 36 hours (11.65%), and 48 hours (16.59%). Pressure sores were reported on the forehead by 53 (19.92%) participants, the nose by 54 (20.30%), the cheek by 31 (11.65%), and behind the ear by 77 (65.71%) participants. The optimal size of PPE was experienced only by 76 (28.57%) participants, while 73 (27.44%) of them felt tight and 117 (43.98%) felt loose.

Conclusions

To minimise discomfort while managing infectious diseases, HCWs can adopt several practices like taking regular breaks, ensuring humane working hours, utilising high-quality PPE, and wearing properly fitting gear. By implementing these measures, HCWs can enhance their ability to handle infectious diseases effectively while prioritising their comfort and well-being.

## Introduction

The pandemic caused by the severe acute respiratory syndrome coronavirus-2 (SARS-CoV-2) has put unprecedented stress and discomfort on front-line healthcare workers (HCWs). Long-term usage of PPE may induce unfavourable symptoms [1], more so in the summer season when facilities like centralised air conditioners are unavailable or are shut down for fear of spreading the infection in tertiary care hospitals. This lack of PPE kit standardisation may negatively affect their ability to perform duties [2]. Thus, clinical efficacy, acceptance of PPE kits, and tolerability by HCWs in the COVID setup were necessary. Only a very limited number of studies on PPE tolerability and its effects on HCWs have been done in India. Hence, the need for the study is to evaluate the clinical effects and tolerability of PPE kits on HCWs while engaged in their daily routine activities in intensive care units (ICU), emergency rooms, and COVID wards.

## Materials and methods

This cross-sectional study was conducted at a tertiary care centre over four months, from May 2021 to August 2021, during the second COVID wave in South India. It consisted of 266 healthcare workers chosen by simple random sampling in the age group between 20 and 50 years using PPE during the COVID-19 pandemic after obtaining ethical committee approval from the Vydehi Institutional Ethics Committee, Vydehi Institute of Medical Science and Research Centre, Bangalore, India (approval number: ECR/747/Inst/KA/2015/RR-18). The personal protective equipment (PPE) used was a non-woven disposable PPE kit that included a pair of nitrile gloves, a pair of shoe covers, a head cover, an N95 mask with a surgical mask, a face shield, goggles, and a gown made of spin-bonded polyester. The HCWs were already trained for donning, doffing, and proper use of PPE as this was the second COVID pandemic wave. Nosocomial infections and standard operating procedures were explained to all healthcare worker participants before treating patients. A comprehensive survey in the form of a physical questionnaire was administered to all participants following the acquisition of written consent (Table 1).

**Table 1:** Questionnaire on the challenges and discomforts faced by healthcare workers in the use of PPE

Questions	Code	Options
I hereby consent to take part in the study and give valid information	1A	Yes
	1B	No
Designation	2A	Physician
	2B	Nurse
	2C	Technician
Age (in years)	3A	20-30 years
	3B	31-40 years
	3C	41-50 years
Gender	4A	Male
	4B	Female
Were you menstruating when you were using PPE?	5A	Yes
	5B	No
	5C	NA
Married	6A	Yes
	6B	No
Department	7A	Emergency
	7B	ICU
	7C	Ward
Were you well informed on standard training on nosocomial infections before treating patients?	8A	Yes
	8B	No
Were you well informed on the standard operating procedure (SOP) for wearing PPE?	9A	Yes
	9B	No
Time taken for donning a suit for PPE	10A	<10 minutes
	10B	10-15 minutes
	10C	15-20 minutes
	10D	>20 minutes
Maximum time spent wearing PPE	11A	4-6 hours
	11B	6-8 hours
	11C	>8 hours
Your maximum tolerance time in PPE	12A	0-2 hours
	12B	2-4 hours
	12C	4-6 hours
	12D	6-8 hours
Number of days PPE was used continuously	13A	1-3 days
	13B	4-7 days
Discomforts felt while wearing PPE	14A	Chest suffocation
	14B	Difficulty in performing intubation
	14C	Difficulty in seeing clearly
	14D	Dizziness
	14E	Excessive sweating
	14F	Micturition desire
	14G	Nausea
	14H	Retroauricular pain
	14I	Stomach burns
	14J	Thirst or dry throat
	14K	Others:___________________
Discomforts felt after doffing PPE	15A	Chest suffocation
	15B	Dark coloured urine
	15C	Dizziness
	15D	Dry mouth
	15E	Dry skin
	15F	Headache
	15G	Reduced ability to concentrate
	15H	Stomach burns
	15I	Tiredness
	15J	Reduced alertness
	15K	Others:___________________
The first thing on my mind after doffing	16A	Drink water
	16B	Eat something
	16C	Clean yourself
	16D	Urination
	16E	Taking rest
	16F	Others:___________________
Misting of goggles	17A	Yes
	17B	No
Pressure sores, if any	18A	Yes
	18B	No
If yes, places where pressure sores are seen	19A	Forehead
	19B	Nose
	19C	Cheek
	19D	Behind ear
	19E	NA
Skin injuries due to gloves	20A	Yes
	20B	No
Time needed to restore after a shift	21A	12 hours
	21B	24 hours
	21C	36 hours
	21D	48 hours
	21E	>48 hours
Were you infected by SARS‑CoV‑2 at work?	22A	Yes
	22B	No
How was the fitting of the PPE?	23A	Loose
	23B	Tight
	23C	Optimal fit

This questionnaire encompassed demographic information, the impact of heat stress, experienced symptoms, and any physical impairments encountered by healthcare workers while wearing personal protective equipment (PPE) amid the COVID-19 pandemic [3]. The PPE, which was supplied by the Vydehi Institute of Medical Science and Research Centre, was obtained from the same vendor and was of the same quality in accordance with the universally accepted protocol.

Inclusion criteria

Doctors (intern medical officers, postgraduates), nurses, and technicians (radiology, laboratory) working in shift duty hours (8 AM-2 PM, 2 PM-8 PM, 8 PM-8 AM) for a minimum of three consecutive days were included. Healthcare workers between the ages of 20 and 50 were included in the study. Written consent was obtained from all the participants, following which the questionnaires were distributed.

Exclusion criteria

Participants with any comorbidities such as diabetes, hypertension, thyroid disorders, medications such as corticosteroids, a recent history of febrile illness (less than one month), or any infections, and females with gynaecological symptoms were excluded. Healthcare workers older than 50 years were not allowed to work in the COVID area by the institution due to possible comorbid complications.

Statistical analysis

Statistical analysis was performed and analysed using IBM Statistical Package for Social Sciences (SPSS) version 19 (IBM Corp., Armonk, New York). The mean and standard deviation, or median, were used to present continuous variables, while frequency and percentage were used to present categorical variables.

Sample size

The sample size was calculated by using n=(Z^2 pq) /d^2 and Z=1.96 at the 95% confidence level as the underlying formula, and the minimum sample size was 246.

## Results

**Figure 1:**
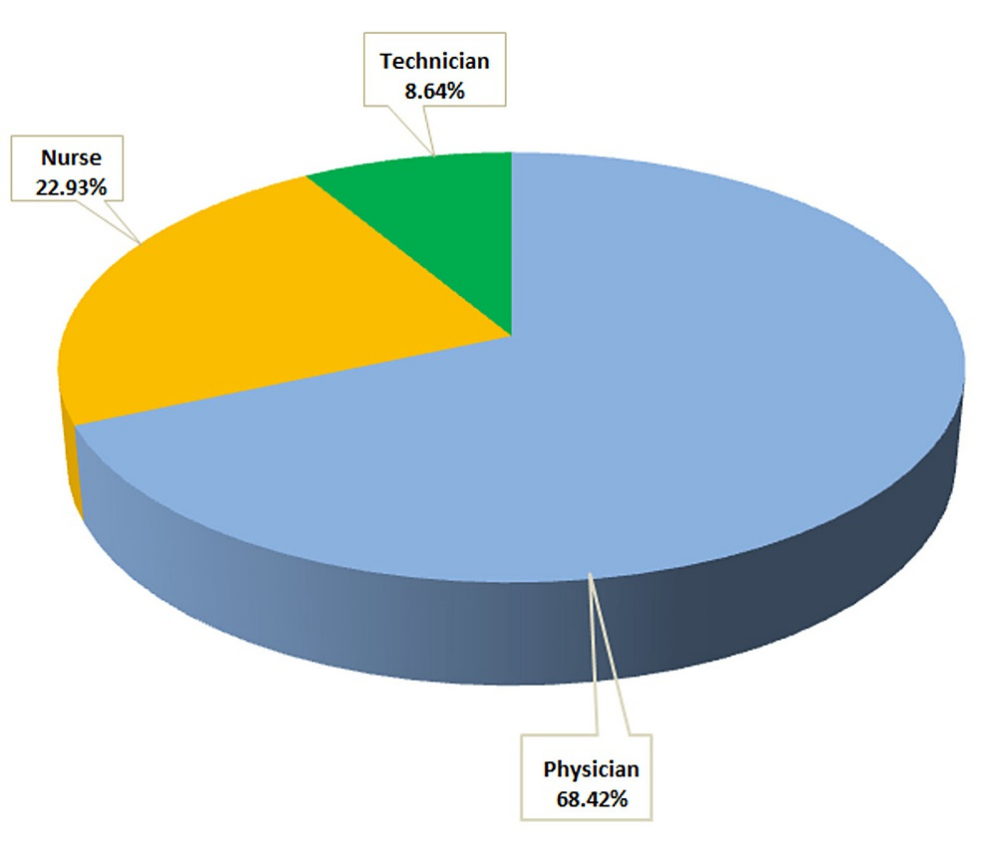
The distribution of healthcare professionals who participated in the study

The research consisted of 266 participants, and their distribution is depicted in the pie chart (Figure 1).

**Figure 2:**
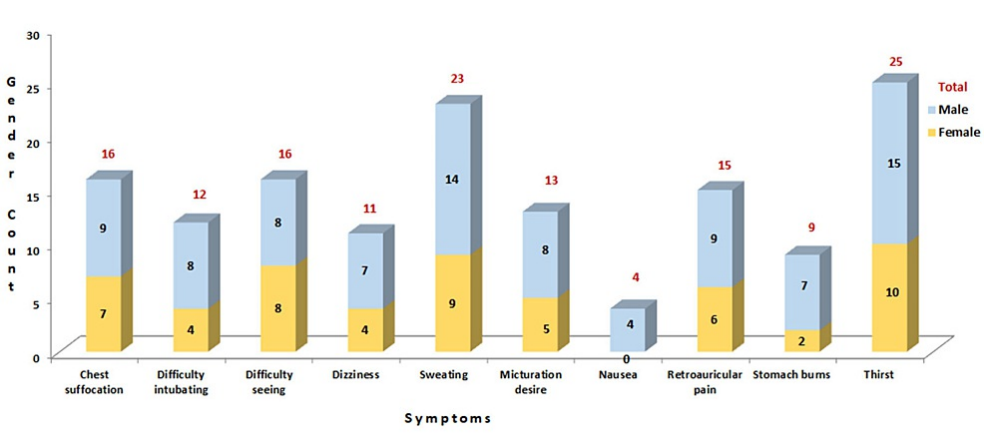
The effects of prolonged PPE use: discomforts experienced during one- to three-day work periods

**Figure 3:**
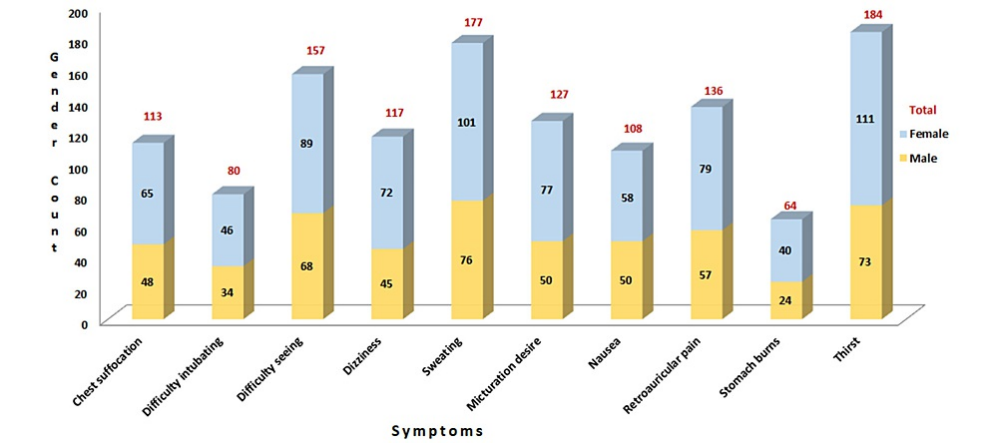
Effects of prolonged PPE use: discomforts experienced during four- to seven-day work periods

Of these respondents, 121 (45.48%) were male and 145 (54.52%) were female. The mean ages of the subjects were 28.18 years with a standard deviation of 5.64 and ranged between 22 and 50 years, of which many HCWs belonged to the 20-30 age group. Eighty-six (32.33%) females reported dysmenorrhoea while wearing the PPE kit, and 65 (24.43%) participants were married. Thirty-five (13.15%) HCWs were posted in the emergency facility, while 82 (30.82%) were in the intensive care unit (ICU), and 149 (56.01%) were posted in the ward. One hundred and ninety-two (72.18%) HCWs completed standard training on nosocomial infection, and 198 (74.43%) HCWs had completed standard operating procedures before treating patients. The time for donning the suit was 10 minutes for 103 (38.72%) HCWs, 10-15 minutes for 117 (43.98%) HCWs, 15-20 minutes for 42 (15.78%) HCWs, and >20 minutes for four (1.50%) HCWs. The maximum time the PPE was worn was four to six hours for 31 (11.65%) HCWs, six to eight hours for 103 (38.72%) HCWs and >eight hours for 132 (49.62%) HCWs. The PPE kit was tolerated as follows: 20 (7.51%) HCWs for 0 to two hours, 68 (25.56%) HCWs for two to four hours, 107 (40.22%) HCWs for four to six hours, and 71 (26.69%) HCWs for six to eight hours; 240 (90.22%) HCWs that used PPE for four to seven days reported more symptoms than those for one to three days (9.77%) (Figures 2-3).

**Figure 4:**
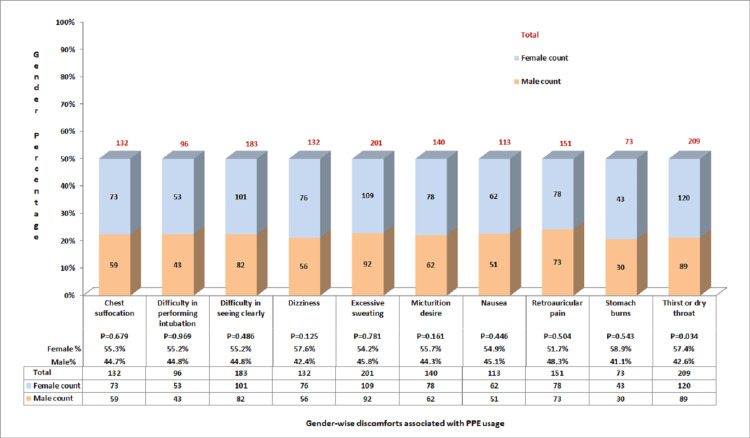
Discomforts associated with PPE use, according to gender

Discomforts experienced by HCWs during the use of PPE illustrated that thirst and dry throat were significant in women when compared to other symptoms like chest suffocation, difficulty in performing intubation due to poor vision, difficulty in seeing clearly due to fogging, dizziness, excessive sweating, micturition desire, nausea, retro auricular pain, and stomachache (Figure 4).

**Figure 5:**
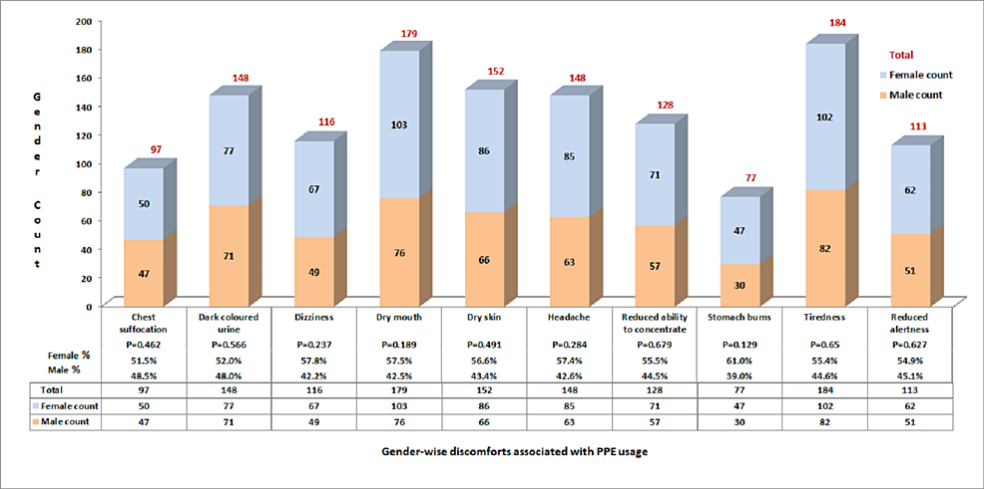
Post-doffing discomforts by gender: exploring challenges associated with PPE usage

The most common discomfort felt after doffing was tiredness and dry mouth due to dehydration, among the spectrum of symptoms assessed (Figure 5).

The first things on the participants' minds after doffing were to clean themselves (163 participants (61.27%)), drink water (182 participants (68.42%)), eat something (96 participants (36.09%)), rest (78 participants (29.52%), and urinate (88 participants (33.88%)) (Table 2).

**Table 2:** The first thing that came to the participants' minds after doffing

First thing that comes to mind after doffing	Male (n=121)	Female (n=145)
Clean yourself	64.46% (78)	58.62% (85)
Drink water	67.76% (82)	68.96% (100)
Eat something	38.01% (46)	34.48% (50)
Take rest	24.79% (30)	33.10% (48)
Urination	36.36% (44)	30.34% (44)

One hundred and seventy (63.90%) HCWs experienced misting of goggles, and 117 (43.98%) HCWs suffered pressure sores on places like the forehead (53 HCWs, 19.92%), nose (54 HCWs, 20.30%), cheek (31 HCWs, 11.65%), and behind the ear (77 HCWs, 65.81%). Also, 81 (30.45%) HCWs suffered skin injuries while wearing gloves. The time taken to restore after a shift for non-COVID ward duty was 12 hours, followed by 24 hours for most of the HCWs (Table 3).

**Table 3:** After-shift restoration time based on gender

Hours required for restoration	Male (121)	Female (145)
12 hours	37.19% (45)	37.93% (55)
24 hours	34.71% (42)	33.79% (49)
36 hours	14.04% (17)	9.65% (14)
=/>48 hours	14.04%(17)	18.62%(27)

Fifty (18.7%) HCWs contracted COVID while doing the COVID rotations. When it came to PPE sizes, optimal size was experienced only by 76 (28.57%) participants, while 73 (27.44%) of them felt it was tight and 117 (43.98%) felt it was loose.

## Discussion

Healthcare workers (HCWs) have raised concerns regarding the wearing of PPE as it poses risks to infection control, including increased susceptibility to heat exhaustion and potential cognitive impairment. [4]. Even though PPE is a formidable physical barrier for the skin and mucous membranes between infections like viral or bacterial ones, it is cumbersome and uncomfortable to wear. Moreover, it restricts free movements and reduces the ability to perceive surroundings, which leads to compromised precision while performing tasks [5].

As we know that PPE must be worn in a sterile and safe setting in order to avoid infection [6], it took most of them around 10-15 minutes to complete donning. Donning requires proper training and strict adherence to protocols in order to prevent contamination, thereby causing a delay in treatment [7]. Due to the rising cases of COVID-19, the HCWs worked more than eight hours for every shift, despite their tolerance being limited to four to six hours due to numerous discomforts when wearing PPE. Long-term exposure to heat and sweat, along with mask metal clip compression, generated a variety of physical discomforts [8]. It is due to the fact that PPE kits trap heat from our bodies due to their impermeable nature, which leads to excessive sweating over prolonged periods of use [4]. The elastic band that attaches to the face shield compresses the mask strings, causing pressure sores [4, 9], particularly behind the ear, which is congruent with the study by Sinu José et al. [8]. This can be prevented to a certain extent by providing good-quality padding and applying a thin layer of petroleum jelly to pressure-sore-prone areas before donning. Headaches were a typical occurrence, according to healthcare personnel [10]. It is attributed to the tight fitting of the goggles, which causes eye strain, and the face masks, which result in poor ventilation.

A week of working eight hours a day made these symptoms worse by taking a toll on their mental stamina and worsening their psychological stress, which led to decreased productivity at work and at home [11]. Our study showed it takes at least 24 hours after a shift for most HCWs to revive. Failure to do so leads to a refusal to work [12]. A 15-minute break every three hours can help the HCWs eat, drink, and relieve themselves [13, 14]. For this method to be successful, an adequate quantity of PPE kits that fit appropriately must be supplied to the HCWs. As the HCWs sweat excessively, they lose more water from their bodies and need to restore the electrolytes they've lost by drinking between breaks [15]. HCWs faced difficulty getting the correct PPE sizes due to shortages, and moreover, the PPE was worn on top of scrubs. Snug PPE is more likely to tear, which can result in the HCWs being exposed to SARS-CoV-2. When walking, slack PPE can tear, which can result in an increased risk of tripping. Waist circumference and height are the two factors that should be taken into account when making recommendations for PPE sizes. It is noted that HCWs worked in wards that were poorly air-conditioned, which led to heat stress more quickly in tropical environments. It appears that HCWs in the ICU were better able to endure their gear because of the air conditioning in the ICU, leading to a higher level of HCW compliance with PPE use.

The PPE kits are also used to treat tuberculosis, Middle East respiratory syndrome (MERS), and influenza. Hence, improving PPE quality can prevent HCWs from becoming infected [16, 17]. A clinical trial had shown that N95 respirators are superior to surgical masks in preventing respiratory virus infection in healthcare workers [18]. However, the scarcity of N95 respirators could have made a few HCWs test positive for COVID-19 due to the use of surgical masks [19]. Although these issues were highlighted as an area of concern during the Ebola virus disease outbreak [20], the same issues are clearly evident during the COVID-19 pandemic. Shortages of PPE have led to concerns internationally due to higher demand during the outbreak [21-25].

According to a meta-analysis, regular hand hygiene was found to have a significant protective effect against influenza infection during the 2009 pandemic. Additionally, facemask use is associated with a non-significant protective effect [26]. As in the 2022 scenario of a global monkeypox outbreak, PPE comfort should be enhanced with better material quality and surplus quantity to suit climatic conditions and availability globally [27, 28]. The idea that PPE can be worn by comorbid immunodeficiency patients to protect against COVID-19 can only happen with improved PPE compliance. Hence, effective use of PPE, the appropriate size of PPE, better material quality, and proper removal and disposal can help reduce the discomforts faced by healthcare workers [29] in all climatic conditions.

The strength of the study highlights the difficulties of functioning in southern India's tropical climate while wearing PPE and the fairly equal distribution between the genders in its findings.

The limitations of this study were that no follow-up was done with regards to HCWs who utilised PPE incorrectly, resulting in HCWs becoming infected with active COVID, and this study was a single-centre study. In addition, the present study does not examine the emotional well-being of PPE users.

## Conclusions

Wearing PPE can cause a wide range of discomfort symptoms that can be alleviated by taking short breaks between shifts by doffing and donning. As demonstrated in this study, the findings can be used to reduce discomfort in the handling of contaminated illnesses that necessitate the use of PPE. Modifications to the design or use, modifying the length of time that PPE is required to be worn during a shift, or providing an appropriate working environment could alleviate some of the discomforts and impaired performance experienced by HCWs when wearing PPE.
